# Reply

**DOI:** 10.1016/j.jacig.2025.100615

**Published:** 2025-11-21

**Authors:** Reina Hara, Yoshito Takeda

**Affiliations:** Department of Respiratory Medicine and Clinical Immunology, Osaka University Graduate School of Medicine, Osaka, Japan

To the Editor:

We sincerely thank Vijayasimha and Jayaswal for their correspondence^1^ regarding our article, “Potential asthma biomarkers identified by nontargeted proteomics of extracellular vesicles in exhaled breath condensate.”^2^ We appreciate their recognition of extracellular vesicles (EVs) as promising breath prints for biomarker discovery and clinical translation in allergy and autoimmunity.^1^

As has been pointed out, EVs provide a unique opportunity to serve as a “liquid biopsy,” encapsulating proteins, cytokines, and nucleic acids that reflect cell-cell communication such as immune activity.^3^ This property has great potential to overcome the limitations of conventional biomarkers such as IgE levels or eosinophil counts, particularly in heterogeneous diseases such as asthma. Our group has previously reviewed the role of EVs in respiratory diseases, highlighting their promise as minimally invasive biomarkers across chronic lung conditions.^4^ That work provided a conceptual framework for the current study.

We agree with Vijayasimha and Jayaswal^1^ regarding their emphasis on methodologic challenges. Standardization of EV isolation and characterization remains essential, and interlaboratory harmonization will be critical for robust validation.^5^ In our study,^2^ we adhered to established International Society for Extracellular Vesicles guidelines and implemented stringent quality controls. Nevertheless, multicenter studies and consensus protocols will be needed to advance reproducibility and translation.

The issue regarding specificity is important. Although some EV-associated proteins may reflect nonspecific airway inflammation, we identified candidate proteins linked to epithelial-immune interactions and type 2 inflammation, lending biologic plausibility to their relevance in asthma. As we emphasized in our article,^2^ independent validation in larger cohorts and with orthogonal assays is a necessary next step.

Finally, our laboratory is actively pursuing multiomics approaches, integrating EV proteomics with transcriptomic and metabolomic data sets to refine disease-specific molecular profiles. Therefore, we believe that combining EV proteomics with cytokine readouts, as well as other omics modalities, will provide a more comprehensive framework for understanding the complex immunopathology of asthma and ultimately refine precision medicine strategies.

## Disclosure statement

Disclosure of potential conflict of interest: The authors declare that they have no relevant conflicts of interest.
